# Supplementary figures and images for: Viral Metagenomics Revealed Sendai Virus and Coronavirus Infection of Malayan Pangolins (Manis javanica)
Source: Viruses. 2019 Oct 24;11(11):979. doi: 10.3390/v11110979 (PMC6893680; doi:10.3390/v11110979)

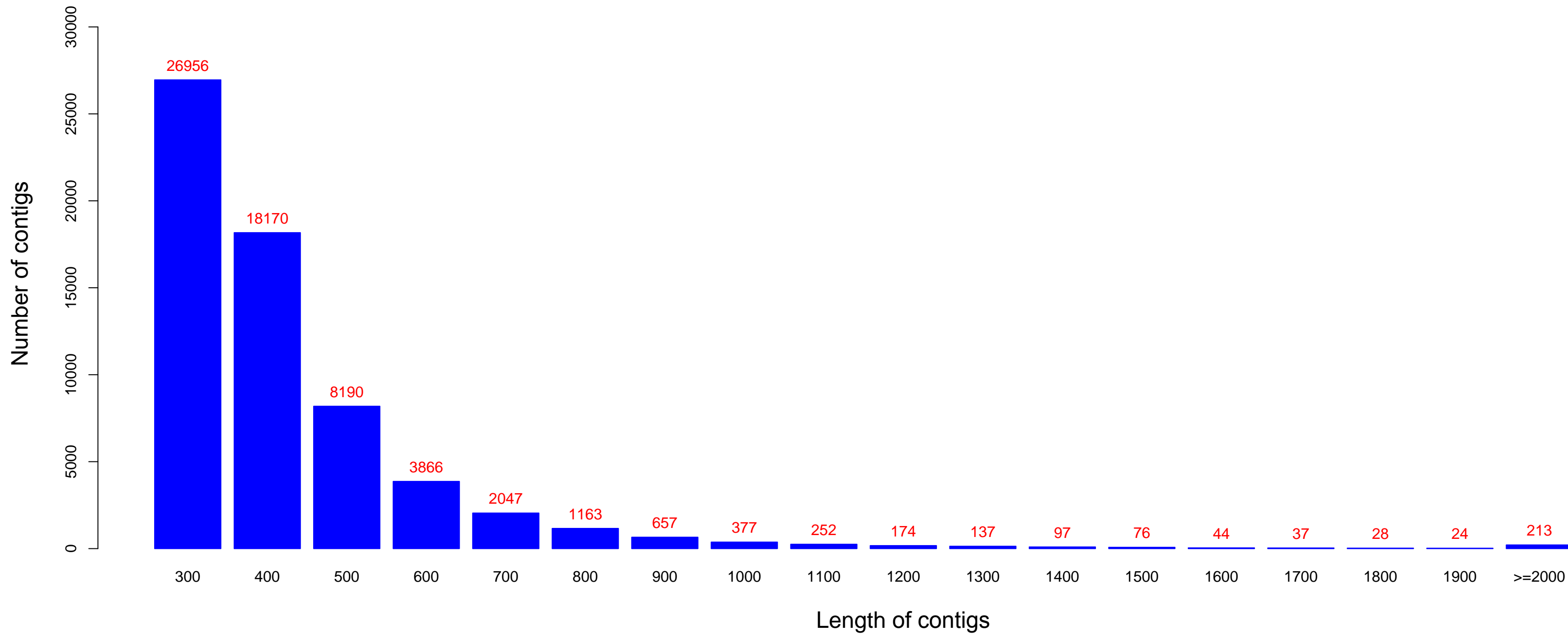

Supplement: Supplementary file 1 [file viruses-11-00979-s001.zip › Figure S1.pdf]
